# Music festival attendees’ illicit drug use, knowledge and practices regarding drug content and purity: a cross-sectional survey

**DOI:** 10.1186/s12954-017-0205-7

**Published:** 2018-01-05

**Authors:** Niamh Day, Joshua Criss, Benjamin Griffiths, Shireen Kaur Gujral, Franklin John-Leader, Jennifer Johnston, Sabrina Pit

**Affiliations:** 1 Harm Reduction and Health Promotion Programs, North Coast Public Health, Mid North Coast Local Health District, PO Box 419, Lismore, NSW Australia; 2 School of Medicine, University of Western Sydney, Sydney, NSW Australia; 3 School of Public Health, Sydney Medical School, The University of Sydney, Sydney, NSW Australia; 4 University Centre for Rural Health, 61 Uralba Street, Lismore, NSW Australia

## Abstract

**Background:**

Drug checking is a harm reduction strategy which allows users to check the content and purity of illicit drugs. Although drug checking has been trialled internationally, with demonstrated value as a harm reduction and health promotion strategy, the use of such services in Australia remains a contentious issue.

This study aimed to investigate the proportion and patterns of illicit drug use among young people, their attitudes towards drug checking at festivals and the potential impact of drug checking on intended drug use behaviour.

**Methods:**

The survey was conducted at a major Australian music festival in 2016. Data was collected from a sample of festival attendees (*n* = 642) aged between 18 and 30 years. A descriptive analysis of the data was performed.

**Results:**

Nearly three-quarters (73.4%) of participants reported that they had used illicit drugs in the past 12 months, most commonly cannabis (63.9%) and ecstasy (59.8%). A large proportion of participants believed ‘somewhat’ or ‘a lot’ that drug checking services could help users seek help to reduce harm (86.5%) and that drug checking services should be combined with harm reduction advice (84.9%). However, two thirds of the participants agreed ‘somewhat’ or ‘a lot’ that drug sellers may use this service as a quality control mechanism (68.6%). Approximately half (54.4%) indicated they would be highly likely and a third (32.7%) would be somewhat likely to utilise free drug checking services should they be available at music festivals. When asked whether the results of drug checking would influence their drug use behaviour, participants reported that they would not take substances shown to contain methamphetamine (65.1%), ketamine (57.5%) or para-methoxyamphetamine (PMA) (58.4%).

**Conclusion:**

The majority of festival attendees aged 18–30 participating in this study reported a history of illicit drug use and were in favour of the provision of free drug checking at festivals. A considerable proportion reported that the results of drug checking would influence their drug use behaviour. The findings of this study can contribute to the debate regarding whether drug checking services could potentially play a major role in harm reduction and health promotion programming for young people attending festivals.

## Background

In the most recent National Drug Strategy Household Survey (NDSHS) in 2016, almost 8.5 million (42.6%) Australians aged 14 years and over reported use of illicit drugs in their lifetime, with 3.1 million (15.6%) reporting use in the last month [1]. Prevalence was highest in the 20–29 years age group, of which 28.2% reported use in the last 12 months [1].

Internationally, music festival attendees report particularly high levels of illicit drug use compared with the general population [2–4]. Consistent with this, studies undertaken at festivals across Australia reflect a considerably higher rate of illicit drug use than is seen in same-age groups in the general population. A cross-sectional study of 1365 adolescents, conducted at a music festival in Australia in 2011, found 52% had used illicit drugs at least once, with 25% doing so in the previous month [5]. Another study conducted from 2005 to 2008 in Australia demonstrated that 44% of music festival attendees had used illicit drugs in the last month [6].

Risks associated with the use of illicit drugs such as methylenedioxymethamphetamine (MDMA (3,4-methylenedioxymethamphetamine) or ecstasy) include adverse side effects such as hyperthermia, seizures, hyponatraemia, rhabdomyolysis and multi-organ failure causing death [7]. In addition, the inclusion of other substances in illicit drugs may also cause significant harm, both through an unexpected increase in purity resulting in overdoses and through the undesired inclusion of additives such as paramethoxyamphetamine (PMA), butylone and methylone [8, 9].

Due to the considerable discrepancy between the prevalence of drug use in populations of festival goers and the general population, it can be theorised that festival goers are more vulnerable to these harmful outcomes and are therefore an important group to be targeted for harm reduction and education.

### Harm reduction

Several organisations in the USA and Europe offer anonymous drug checking services to the public [10]. Countries such as France and Spain, which have a comparable legal stance on drug use to Australia, have active drug checking services available as a harm reduction intervention by communities and local governments [11, 12]. On-site drug checking interventions are already in place in the Netherlands and Austria [13]. Dutch citizens have been able to test their illicit drugs at government-funded Drug Information and Monitoring System (DIMS) facilities since the 1990s, for the purposes of harm reduction and prevention [14]. Utilisation of these services has been demonstrated to influence user behaviour [15]. The Trans European Drug Information (TEDI) program saw drug checking being offered at a number of festivals across Europe, helping to profile the global drug market and creating a number of harm reduction avenues that are aligned with drug checking [16]. Substance education, psychosocial counselling and referral options can be offered to an at-risk population while drug analysis is being performed [17]. Drug checking has led to an awareness of circulating harmful substances, which has resulted in a decline of these products on the market in subsequent years [2, 18, 19]. It may also allow for the implementation of early warning systems to inform drug users of dangerous substances before additional harm can result [2, 16].

However, some concerns exist regarding drug checking being perceived as encouraging drug use and the limitations of accurate substance detection of reagent testing kits [20]. These kits are limited in the number of substances they can identify, are operator-dependent and do not identify non-drug components [21]. On-site laboratory-grade testing, as used in the CheckIt program in Austria [17], provides more accurate and detailed analyses for consumers about a wider range of substances. There is also the perception that drug checking could act as a quality control mechanism for drug dealers [22].

### Community perceptions

Research commissioned by the Australian National Council on Drugs found that more than 82% of a sample of 2335 Australians aged between 16 and 25 years were in favour of the introduction of drug checking [23].

Despite public support and backing from numerous prominent political and law enforcement figures [24, 25], legislation for drug checking at festivals has yet to be passed by any state government in Australia. The main arguments against drug checking are that there is a lack of evidence to support its efficacy and that it may appear that the government is condoning drug use [26]. However, a motion in August 2016 passed unopposed through the Australian Senate calling for the introduction of evidence-based harm reduction policies to counteract harmful drug use, including the cessation of sniffer dog use at festivals and implementation of drug checking trials [25].

This study aims to increase our understanding of attitudes and behaviours towards drug checking services among music festival patrons. To our knowledge, this is the first study based at a music festival to survey attendees about drug checking services in Australia and will provide valuable knowledge that will contribute to the ongoing debate surrounding drug testing.

## Methods

### Survey development and outcome measures

The survey content and structure was guided by previous research in the area [1, 13, 27] and members of the research team with expertise in the fields of drug and alcohol, sexual health, health promotion and public health. All survey respondents were asked about their demographics, illicit drug use and attitudes towards drug checking services. Those who indicated a history of illicit drug use were further questioned about their attitudes and concerns towards drug content and purity, attempts to find out about the content and purity of drugs they had previously taken and the likelihood of using a free drug checking service.

The data collected was predominately quantitative, with the exception of one open-ended question asking drug users why they had never attempted to find out about the content/purity of their drugs (if applicable). Two waves of pilot-testing of the survey were conducted with groups of young adults (*n* = 10 and *n* = 12, respectively) who were representative of the target audience, with the data collection tool refined after each wave.

### Recruitment and data collection

The data collection for this study was undertaken over 2 days at a major Australian popular music festival as part of an established sexual health promotion stall. Participants were recruited based on their estimated age being within the target range of 18–30 years. Participants were given a participant information statement and were informed about what the survey involved and the purpose of the study. Completion of the survey was taken as consent. Participants completed the paper-based survey anonymously and independently and placed the survey in a closed box to ensure confidentiality. No identifying information was collected to ensure anonymity of participants. Participants were only approached during daylight hours to minimise the likelihood of participants being intoxicated at time of completion and were not eligible to participate if they were visibly intoxicated. No incentives were provided. The surveys were provided by independent researchers not linked to the health promotion stall, who were available to answer participants’ questions if required. Participants were given a short summary of what drug checking is and the information it can provide.

### Data analysis

Analyses were conducted using SPSS Version 22. Chi-square tests were conducted to determine associations between categorical variables and *t* test for continuous variables. A *p* value of < 0.05 was considered to be statistically significant. Missing data was treated by excluding the participant from that question.

The open-ended question was coded thematically to identify reasons for not attempting to find out about drug content and purity. Initial codes were created based on a review of the complete data set by two researchers, who then created the coding scheme through discussion and reaching consensus. This coding scheme was then applied to the data.

## Results

### Socio-demographic and drug use characteristics

A total of 642 people between the ages of 18 and 30 years participated in the survey. The majority of survey responders were female (60.5%), aged 18–21 years (62.2%), single (56.8%), heterosexual (90.4%), part-time employed (52.6%) and/or studying full-time (48.9%). A disproportionate number of females were surveyed, although chi-square testing failed to demonstrate a statistically significant difference in the prevalence of drug use in the past 12 months between males and females (*p* = 0.060). Most participants reported having used alcohol (97.8%) at least once during their lifetime or illicit drugs (73.4%) at least once during the last 12 months. The drugs most commonly used in the previous 12 months were cannabis, ecstasy and cocaine (63.9, 59.8 and 34.1%, respectively) (see Table 1).

**Table 1:** Drug use in the last 12 months (*N* = 642)

Drug	%
Cannabis (%)	63.9
Ecstasy/MDMA (%)	59.8
Cocaine (%)	34.1
Hallucinogens (%)	20.2
Amphetamine (%)	18.4
Illicit pharmaceuticals (%)	13.6
Ketamine (%)	12.5
Inhalants (%)	8.4
Synthetic cannabis (%)	4.8
Methamphetamine (%)	4.7
GHB (%)	2.3
Steroids (%)	1.7
None (%)	26.6

### Attitudes towards drug checking services

All survey respondents were asked about their attitudes towards drug checking services. The majority of participants agreed ‘somewhat’ or ‘a lot’ that drug checking services should be provided for free at festivals (86.2%), with a slightly lower proportion agreeing ‘somewhat’ or ‘a lot’ that if the services were not provided for free that it should be provided at cost (67.5%). A large proportion of participants believed ‘somewhat’ or ‘a lot’ that drug checking services could help users seek help to reduce harm (86.5%) and that drug checking services should be combined with harm reduction advice (84.9%). However, two thirds of the participants agreed ‘somewhat’ or ‘a lot’ that drug sellers may use this service as a quality control mechanism (68.6%). When the attitudes of those who had used drugs in the past 12 months were compared to those who had never used drugs (Table 2), drug users were found to be significantly more likely to believe that drug checking services should be provided for free (*p* = 0.019), could assist in drug users seeking help to reduce harm (*p* = 0.019) and that the services should be combined with harm reduction advice (*p* = 0.020).

**Table 2:** Attitudes towards drug testing

	Non-drug users	Drug users
Drug checking services should be provided for FREE at festivals (*n* = 639)		
Not at all/A little	19.4 (33)	11.7 (55)
Somewhat/A lot	80.6 (137)	88.3 (414)
If not free, drug checking services should be provided AT COST at festivals (*n* = 631)		
Not at all/A little	34.1 (56)	31.9 (149)
Somewhat/A lot	65.9 (108)	68.1 (318)
Drug checking services could help users seek help to reduce harm (*n* = 629)		
Not at all/A little	18.9 (31)	11.6 (54)
Somewhat/A lot	81.1 (133)	88.4 (411)
Drug checking services should be combined with harm reduction advice (*n* = 628)		
Not at all/A little	20.7 (34)	13.1 (61)
Somewhat/A lot	79.3 (130)	86.9 (403)
Drug sellers may use the service as a quality control mechanism (*n* = 622)		
Not at all/A little	35.8 (58)	29.8 (137)
Somewhat/A lot	64.2 (104)	70.2 (323)

Each question was checked for responses by gender, with a significant difference found in the question regarding the provision of at-cost drug checking services, demonstrating that a significantly higher proportion of males (73.5%) selected ‘somewhat’ or ‘a lot’ compared with females (64.0%) (*p* = 0.013). The remaining questions in this table failed to demonstrate a difference between genders.

### Attitudes, beliefs and drug checking behaviour of drug users

Those who indicated a history of illicit drug use (*n* = 471) were asked additional questions about their attitudes, beliefs and behaviours regarding drug use and checking (Table 3). A significantly higher proportion of men (61.1%) reported they were ‘highly likely’ to use a free drug checking service compared with women (48.0%) (*p* = 0.018).

**Table 3:** Attitudes of drug users towards testing

	Total % (*n*)
Concern about drug content and/or purity (*n* = 460)	
Not at all/A little	47.2 (217)
Somewhat/A lot	52.8 (243)
Attempts to find out about drug content and/or purity (*n* = 462)	
Never	37.2 (172)
Occasionally	29.0 (134)
Often	19.5 (90)
Always	14.3 (66)
Likelihood of using a free drug checking service (*n* = 447)	
Not likely at all	13.0 (58)
Somewhat likely	32.7 (146)
Highly likely	54.4 (243)
What methods have you used to attempt to find out about the content and purity of illicit drugs you take? (*Multiple answers possible*) (*n* = 453)	
Friends	78.6 (356)
Dealers	36.2 (164)
Websites	35.8 (162)
Personal previous experience	43.5 (197)
Personal use of drug testing kit	5.7 (26)
Other’s use of drug testing kit	6.4 (29)
None	13.9 (63)

Out of 172 participants who had never attempted to find out about drug content and/or purity, 137 reported reasons for not doing so. Qualitative analyses revealed that the most common reasons for not attempting to find out the content and purity of drugs included having limited access and knowledge about drug checking services (*n* = 59; 43.1%), lack of concern (*n* = 41; 29.9%), the belief that drugs were from a reliable source (*n* = 18; 13.1%), infrequent drug use (*n* = 14; 10.2%) or a primary use of non-synthetic drugs such as marijuana (*n* = 5; 3.6%).

### Potential impact of drug checking services on drug use

Those who indicated a history of illicit drug use (*n* = 471) were also asked whether the results of a drug check would influence their decision to take the drug. The majority reported that they would not take their drug if it contained any of the chemicals mentioned in Table 4 (with the exception of ecstasy). A relatively high level of data was missing from this question; however, no significant differences were found between those who completed the question and those who did not in terms of age or gender.

**Table 4:** Proportion of participants that would still take a drug if testing showed it contained the additional presence of the drugs listed

Drug	Would take	Would not take	Do not know
Ecstasy/MDMA % (*n* = 422)	69.7 (294)	17.8 (75)	12.6 (53)
Amphetamine % (*n* = 398)	32.9 (131)	46.7 (186)	20.4 (81)
Methamphetamine % (*n* = 390)	14.9 (58)	65.1 (254)	20.0 (78)
Ketamine % (*n* = 398)	20.9 (83)	57.5 (229)	21.6 (86)
DXM % (*n* = 388)	7.0 (27)	59.3 (230)	33.8 (131)
2C-B/CA % (*n* = 390)	7.9 (31)	57.4 (224)	34.6 (135)
DOB % (*n* = 386)	3.1 (12)	59.1 (228)	37.8 (146)
DOI % (*n* = 384)	3.1 (12)	58.6 (225)	38.3 (147)
Methylone % (*n* = 381)	3.1 (12)	59.3 (226)	37.5 (143)
Butylone % (*n* = 380)	1.8 (7)	59.2 (225)	38.9 (148)
Naphyrone % (*n* = 377)	1.9 (7)	59.4 (224)	38.7 (146)
Opiates % (*n* = 383)	15.7 (60)	53.3 (204)	31.1 (119)
PMA/PMMA % (*n* = 365)	3.8 (14)	58.4 (213)	37.8 (138)
Other % (*n* = 315)	2.5 (8)	59.4 (187)	38.1 (120)
No reaction/benign substances % (*n* = 328)	6.4 (21)	57.6 (189)	36.0 (118)

*DXM* dextromethorphan, *2C-B/CA* 2,5-dimethoxy-4-bromophenethylamine, *DOB* dimethoxybromoamphetamine, *DOI* 2,5-dimethoxy-4-iodoamphetamine

## Discussion

This study investigated the prevalence of illicit drug use among young people attending a large Australian music festival, their attitudes towards on-site drug checking and the potential impact on-site drug checking would have on drug taking behaviour. Consistent with previous research in Australia and internationally [3, 5, 6, 27], the results demonstrate high levels of illicit drug use among this sample of festival attendees, with the rate of illicit drug use being almost three times higher (73.4 vs 28.2%) among festival attendees than in the young adult Australian population (20- to 29-year-olds) [1].Of particular note are the differences in the prevalence of cannabis (63.9 vs 22.1%), ecstasy (59.8 vs 7.0%) and cocaine (34.1 vs 6.9%) in the current study compared to the Australian general population [1]. A Danish study also reported high drug use among festival patrons. Illicit drug use was higher than that found in our study, with 92.8% having used cannabis in the past year, 66.7% having used MDMA and 51.2% having used cocaine in the past year [28].

The majority of study participants were in support of on-site drug checking being available (both free and at cost, 86.2 and 67.5%, respectively) and believed that it would help users seek help to reduce harm (84.9%). Free drug checking services were preferred to those at cost, especially among women. While these findings may suggest that charging drug users for drug checking services would be a barrier to access, a recent study into drug service utilisation demonstrated that two thirds of respondents were willing to pay up to $10 for a festival or nightlife drug checking service and almost all were willing to pay up to $5 [29].

Two thirds of participants believed that drug dealers may use the service as a ‘quality control mechanism’. Participants could have interpreted this in two ways. First, dealers could use the service to ensure that their product does not include unintended substances. Second, dealers could use it to promote their product as having a higher amount of a drug and thus being of better quality. Therefore, it is not clear from our survey if this is of concern to the population surveyed; however, it has been identified as an issue elsewhere. In the USA, this is a significant concern and is overcome by organisations providing drug checking services displaying the results of their tests as ratios, rather than quantitative amounts [22]. Due to the amount of unmeasured and undetectable substances in a drug, ratios cannot be linked to quantitative amounts [22]. This allows drug users to be informed about the possible unintended substances in their drugs but not about the quality. Similarly, across many countries in Europe, procedures have been implemented at drug checking services to ensure pills presented by obvious drug dealers are not being analysed, with the identification of drug dealers presumably being based on multiple presentations and high volumes of drugs to be tested [12].

Over a third of drug users in the sample had never attempted to find out about the content and purity of their drugs. A number of reasons were cited for responders not seeking further information, the most common being limited access and knowledge regarding availability of drug checking services. Among the two thirds of people who had attempted to find out about the content and purity of their drugs, only a limited number (5.7%) had used a personal drug testing kit. This highlights the need for further education among drug users regarding the benefits and limitations of reagent drug checking. When asked what methods participants had used to attempt to find out about the content and purity of illicit drugs, 78.6% had used friends, 36.2% had used dealers and 35.8% had used websites. On the contrary, Barratt and colleagues [29] found that 75% of Australian festival and nightlife attendees who had completed a web-based survey had used friends, 63% had used dealers and 53% had used websites.

The vast majority of respondents stated they were somewhat or highly likely to use a drug checking service at a festival (87.1%). This is consistent with recent research [29]. Barratt et al. [29] found that 94% of people would use drug checking service located at festivals or clubs. This supports the theory that drug checking services would potentially be utilised at festivals if offered. However, Barratt and colleagues also found that that 94% would not utilise the service if there was a chance of being arrested and two out of three also would not use the service if they would not be provided with individual results.

This study suggests a proportion of drug users would alter their drug taking behaviour if drug checking revealed certain unexpected or undesired substances in their products. This is consistent with previous work in the area, such as a review of the Austrian CheckIt! Program which indicated that, after testing revealed an unexpected substance, two thirds of people would not take their drug and would go on to warn their friends [17]. An Australian study also found that three quarters of regular ecstasy users reported that they would not take a pill found to contain ‘unknown substances’ [30]. The evidence thus suggests that the outcomes of drug checking has the potential to change drug use behaviour and therefore may reduce harm among this group. However, a number of participants in our study reported that they ‘did not know’ if they would still take a drug upon learning of unintended contents in their product (12.6–38.9% depending on the substance). This finding highlights the need for continued education in this area of public health and the opportunity created through the provision of drug checking services. This is evidenced by the CheckIt! Program demonstrating higher levels of engagement in education opportunities when concurrent chemical drug analysis was being performed [17]. Indeed, Hungerbuehler and colleagues [3] also concluded in their study among drug checking services users in Switzerland that drug checking in combination with a consultation were a vital harm reduction and prevention tool that reached a high-risk group of people who used high doses of drugs frequently and polydrug users. Importantly, the authors also acknowledged that different prevention measures need to be tailored to local situations and different target groups.

Research has identified that current drug checking kits are subjective and difficult to interpret [21], thus providing a precedent for testing being performed by a trained team member, who is well versed in explaining the results. However, the risk of reagent tests not detecting harmful substances remains.

Further research in this area include the feasibility and practicality of using laboratory-grade testing equipment on-site compared to reagent testing. Longitudinal research investigating the long-term effects of drug checking could also help to highlight the potential benefits and risks from the health intervention and education that a drug checking service offers.

### Limitations

The results of our research are based on a convenience sample of music festival attendees, and as such, survey respondents are not likely to be representative of the general population. The predominance of female respondents does not correspond with national data demonstrating that males typically consume more illicit drugs than females [1], potentially limiting the generalisability of this study. The high proportion of females surveyed (60.5%) may also skew the interpretation of the results, given the known differences in drug use prevalence between genders [1]. Testing failed to show a difference in drug use prevalence between genders among our study participants, but other inherent differences may exist. Any interpretation of our results should bear in mind the potential for gender bias.

Self-report was also a study limitation. To reduce the likelihood of safety hazards and inaccurate reporting associated with intoxication of survey participants, surveys were only conducted during daylight hours and participants who looked visibly intoxicated were excluded from participation. This may lead to a sampling bias as a proportion of the population of interest were excluded from participation. Convenience sampling could also lead to systematic bias when comparing the findings to other populations of drug users and to the general population. However, drug use data in our study are comparable with other festivals [3, 5, 6, 12, 16].

Participants were asked about drug use in the last 12 months, but not about frequency of drug use, which may have provided valuable information about a specific subgroup of participants. Grouping once-only drug users with frequent drug users may account for the significant difference between drug users’ and non-drug users’ attitudes towards provision of drug checking services at festivals. However, the purpose of this research was to determine whether drug users would utilise drug checking services at festivals, and as such, it is important that all drug users are considered, regardless of frequency of use.

The missing data about drug checking services and whether or not participants would use drugs containing unintended substances may impact on the validity of the findings from this particular question. A reason for this may be that the question appeared at the end of the survey and participants may have been less likely to complete the survey in its entirety. However, no significant differences were found between responders and non-responders in age and gender.

Questions relating to drug checking services and corresponding behavioural changes are dependent on peoples’ knowledge of harmful substances, the severity of adverse effects and people’s understanding of the accuracy of testing kits. Lack of knowledge may skew results in favour of positive behavioural change.

A major strength of this study is that it is, to our knowledge, the first large survey performed at a music festival that measured attitudes and behaviours towards on-site drug checking services. Reducing harm from illicit drug use is a pertinent social issue, and our research provides valuable insight into the attitudes and behaviours of an at-risk population’s potential use of drug checking services.

## Conclusion

Drug-related harms continue to be of concern at Australian music festivals. Drug checking services have been implemented internationally as a method of harm reduction. The majority of festival attendees reported a history of illicit drug use and were in favour of the provision of both free and at-cost drug checking at festivals. Drug users stated that they would utilise free on-site drug checking services and that the results of these tests would influence their drug-taking behaviours. This has the potential ability to directly minimise harm from ingestion of unintended toxic substances. The findings of this study can contribute to the current debate in Australia regarding whether drug checking services could play a role in harm reduction and health promotion programming for young people attending festivals.

## Abbreviations

2C-B/CA: 2,5-Dimethoxy-4-bromophenethylamine
DIMS: Drug Information and Monitoring System
DOB: Dimethoxybromoamphetamine
DOI: 2,5-Dimethoxy-4-iodoamphetamine
DXM: Dextromethorphan
GHB: Gamma hydroxybutyrate
MDMA: 3,4-Methylenedioxymethamphetamine
NDSHS: National Drug Strategy Household Survey
PMA: Paramethoxyamphetamine
TEDI: Trans European Drug Information
